# Impact of antiretroviral therapy (ART) duration on ART adherence among men who have sex with men (MSM) living with HIV in Jinan of China

**DOI:** 10.1186/s12981-022-00482-z

**Published:** 2022-11-24

**Authors:** Kedi Jiao, Meizhen Liao, Guangmei Liu, Yanmei Bi, Xiuhong Zhao, Qian Chen, Jing Ma, Yu Yan, Chunxiao Cheng, Yijun Li, Wenwen Jia, Lina Wang, Yanwen Cao, Zhonghui Zhao, Xuan Yang, Jing Meng, Jianzhuo Li, Xinrui Li, Chunmei Wang, Dianmin Kang, Wei Ma

**Affiliations:** 1 Department of Epidemiology, School of Public Health, Cheeloo College of Medicine, Shandong University, 44 West Wenhua Road, Jinan, 250012 Shandong People’s Republic of China; 2 Institution for AIDS/STD Control and Prevention, Shandong Center for Disease Control and Prevention, 16992 Jingshi Road, Jinan, 250014 Shandong People’s Republic of China; 3 Shandong Public Health Clinical Center, 12 East Martyrs Mountain Road, Jinan, 250132 Shandong People’s Republic of China; 4 Institution for AIDS/STD Control and Prevention, Jinan Center for Disease Control and Prevention, 2 Weiliu Road, Jinan, 250021 Shandong People’s Republic of China

**Keywords:** Antiretroviral therapy adherence, Antiretroviral therapy duration, Men who have sex with men, China

## Abstract

**Background:**

Consistent and complete adherence is considered an essential requirement for patients on antiretroviral therapy (ART). This study aimed to evaluate the impact of ART duration on ART adherence, identify the trend of complete adherence, and compare the factors associated with ART adherence between short-term and long-term ART group among men who have sex with men (MSM) living with HIV in Jinan of China.

**Methods:**

MSM living with HIV aged 18 or above and currently on ART were recruited from October to December 2020 using convenience sampling. Univariate and multivariable logistic regressions were used to evaluate the impact of ART duration on adherence and compare factors associated with ART adherence between subgroups. The Mann–Kendall test was used to identify the trend of complete adherence.

**Results:**

A total of 585 participants were included in analysis, consisting of 352 on short-term ART (ART initiation ≤ 3 years) and 233 on long-term ART (ART initiation > 3 years). Significant difference of complete ART adherence between short-term and long-term ART group was detected (79.8% vs. 69.1%, *P* = 0.003). Multivariable analysis showed that men with longer ART duration were less likely to report complete ART adherence (AOR = 0.88, 95% CI 0.81–0.95). A descending trend of complete adherence was identified (Z = 1.787, *P* = 0.037). Alcohol use and lack of medication reminders were barriers to complete adherence for both of the subgroups.

**Conclusions:**

Sustained efforts to encourage maintaining adherence for a lifetime are necessary, especially for those on long-term ART. Future interventions should be tailored to subgroups with different ART duration and individuals with specific characteristics.

**Supplementary Information:**

The online version contains supplementary material available at 10.1186/s12981-022-00482-z.

## Background

Despite remarkable progress in HIV prevention and treatment, there were still 1.7 million newly HIV-infected people in 2019 and 38 million people were living with HIV globally [1]. About 23% of new adult HIV infections in 2019 were men who have sex with men (MSM) [1]. In China, there were 1,045,000 reported cases of HIV by the end of October 2020 [2]. Similarly, MSM accounted for 23% of new infections in 2019 [3]. Though MSM only constituted small proportions of the general population, they are at increased risk of acquiring HIV infection.

The introduction of combination antiretroviral therapy (ART) has dramatically reduced morbidity and mortality of people living with HIV (PLWH) [4]. Consistent and complete adherence is considered an essential requirement for PLWH on ART to avoid treatment failure, disease progression and realize its life-extending benefits. However, maintaining a high level of adherence remained a challenge in some regions. For example, a meta-analysis in China showed that the mean rate of > 95% ART adherence dropped from 81.1% at 1 week to 68.3% at 3 months or longer [5]. A broad range of factors associated with ART adherence have been reported including individual-related factors (e.g., understanding of the adherence), treatment-related factors (e.g., adverse drug reactions), psychosocial factors (e.g., depression and stigma) and structural factors (e.g., health workers’ attitudes) [6, 7]. However, these factors usually differed across regions and populations due to different demographic characteristics, culture and availability of resources.

As HIV cannot be eradicated yet, it is essential to know whether PLWH can maintain ART adherence over time. A study in UK and a bi-regional cohort in sub-Saharan Africa and Asia found that the risk of suboptimal adherence decreased with ART duration extended [8, 9]. On the other hand, some prospective studies have reported the adherence to ART declined over time [10–13]. However, most of these studies had short follow-up time (< 24 months), had moderate sample size (< 200), and were conducted in high-income countries (e.g., USA and Canada) before 2010. Studies conducted in different regions, having different observation periods, or measuring adherence using different methods provided conflicting results.

In China, the National Free Antiretroviral Treatment Program (NFATP) was initiated in 2002 and then scaled up rapidly [14]. Following WHO guidelines, China regularly updated the national ART guidelines and started providing free ART to all PLWH regardless of their CD4 levels in June 2016 [15]. Considering that standards of ART initiation and related policies changed over time, it was necessary to understand whether PLWH with different ART duration reported consistent adherence. However, to our knowledge, few studies in China examined the association between ART duration and adherence among PLWH, especially among MSM.

Therefore, this study aimed to evaluate the impact of ART duration on ART adherence, identify the trend of complete adherence based on different ART durations, and compare the factors associated with ART adherence between those on short-term (i.e., ≤ 3 years) and long-term (i.e., > 3 years) ART among MSM living with HIV in Jinan of China.

## Methods

### Setting and participants

This survey was conducted in an infectious diseases hospital in Jinan, Shandong Province of China from October to December in 2020. The hospital serves more than 2000 PLWH, accounting for more than 90% of PLWH in this city. Among them, MSM patients accounted for about 80%. According to ART guidelines in China, all patients need to visit the health care providers in the designated hospital at least every 3 months for medication refilling and related testing (e.g., blood and urine testing).

All eligible patients waiting for their medical visits were invited to participate in this study. Inclusion criteria included: (1) aged 18 years or above; (2) HIV-diagnosed positive and currently on ART in this hospital; (3) born biologically male; and (4) having ever had anal sex with men.

### Survey procedures and data collection

The participants were recruited from the sexually transmitted infections (STI) outpatient clinic of the hospital where PLWH visited regularly for medication refilling using convenience sampling from October 19 to December 4 in 2020. Patients seeking medical visits were referred to a waiting room by the physicians and invited to participate in this study. Those who were interested received a detailed introduction of this study by trained investigators. Those who met the inclusion criteria were asked to provide written informed consents and complete a self-administrated questionnaire. When a questionnaire was completed, investigators checked immediately to identify potential logical errors and verify with the participants. All eligible participants were reimbursed 50 Chinese Yuan (CNY) (about 7.47 USD) after the verification was completed.

The self-administrated questionnaire was developed based on literature review and revised by local STI experts. The information collected included: (1) socio-demographic and risk behavioral characteristics of participants, including age, household, monthly income, education, occupation, marital status, alcohol use, drug use, and medical insurance, etc.; (2) disclosure of sex orientation and HIV status; (3) situation of ART, including year of HIV diagnosis, year of ART initiation, whether having medication reminders, whether reporting side effects, schedule and frequency of daily medication-taking, and etc.; (4) 12-item ART medication knowledge; (5) HIV treatment adherence self-efficacy, which was measured by the 12-item HIV-ASES-Chinese-Version [16]; and (6) ART medication adherence in the past month, which was measured using two questions including “How many times have you missed ART medications in the past month” and “how often did you take medications within one hour of prescribed time in the past month”. Those who took all medications within one hour of prescribed time were defined as “complete adherence”. Otherwise, men were defined as “suboptimal adherence”.

### Data management and statistical analysis

All data were double entered with Epidata 3.1, and discrepancies were checked against the raw data. Descriptive analysis was conducted to compare differences between short-term ART group (i.e. ART initiation ≤ 3 years) and long-term ART group (i.e. ART initiation > 3 years) using chi-square tests or student’s t tests. The grouping was based on current Chinese national ART guidelines released in 2016 that started providing free ART to all PLWH regardless of their CD4 levels [15]. First, a series of univariate logistic regressions were used to determine the association between the independent variables (ART duration as continuous variable and potential covariates) and outcome variable (ART adherence). We included  sex role, sex orientation and sex orientation disclosure in univariate regressions because these characteristics may be mediated by psychosocial and behavioral factors influencing ART adherence [17, 18]. Then variables with *P*-values < 0.1 in the univariate regressions were eligible for entry into the multivariable logistic regression, where adjusted odds ratio (AOR) with 95% confidence intervals (CIs) and two-side *P*-values were calculated. Stepwise method was used in multivariable analysis to select variables and reduce the effect of collinearity. Simultaneously, one-side Mann–Kendall test was used to identify the trend of proportions of PLWH with complete ART adherence. In addition, subgroup analysis was conducted to compare the factors associated with ART adherence between short-term and long-term ART group.

Data management and descriptive analysis was conducted by SPSS 24.0. The logistic regressions and Mann–Kendall test were conducted by R 4.0.4.

### Ethics approval

The study was conducted in accordance with the Declaration of Helsinki. This study was approved by the Ethical Review Committee of School of Public Health in Shandong University (20190210). The written informed consents were obtained from all participants.

## Results

### Descriptive analysis

A total of 586 MSM living with HIV were approached and 585 valid questionnaires were collected, including 352 (60.2%) on short-term ART and 233 (39.8%) on long-term ART. The mean age of participants was 34.38 years. About 60% of participants had an education level of college and above, and about half of participants earned ≤ 5000 CNY every month. Most participants (81.5%) were currently unmarried and 87.8% of participants had medical insurance. The proportion of sex orientation disclosure and HIV status disclosure was 55.7% and 62.2%, respectively. More than half of participants (58.3%) reported alcohol use in the past 12 months, while most of them (93.5% and 90.5% respectively) did not report ever drug use and condomless anal sex in the past 3 months. The proportion of employment in short-term ART group and long-term ART group was 90.1% and 95.7%, respectively. Among short-term ART group, homosexual and bisexual participants accounted for 56.0% and 29.0%, while in long-term ART group, this proportion was 66.1% and 23.2%. More details were displayed in Table 1.

**Table 1:** Comparison of socio-demographical and behavioral characteristics of MSM living with HIV on short-term and long-term ART in Jinan of China (N = 585)

Variables	Total (N = 585)	Short-term ART group (n = 352)	Long-term ART group (n = 233)	χ^2^/t	*P*-value
Age	Mean = 34.38, SD = 8.89	Mean = 32.76, SD = 8.88	Mean = 36.84, SD = 8.35	− 5.570	< 0.001
Living area				0.038	0.845
Urban area	532 (91.1)	320 (90.9)	212 (91.4)		
Rural area or county town	52 (8.9)	32 (9.1)	20 (8.6)		
Education				0.240	0.624
High school and below	277 (38.9)	134 (38.1)	93 (40.1)		
College and above	357 (61.1)	218 (61.9)	139 (59.9)		
Employment				6.306	0.012
Yes	540 (92.3)	317 (90.1)	223 (95.7)		
No	45 (7.7)	35 (9.9)	10 (4.3)		
Monthly income (CNY)				4.635	0.099
≤ 5000	281 (48.0)	178 (50.6)	103 (44.2)		
5001–8000	179 (30.6)	96 (27.3)	83 (35.6)		
> 8000	125 (21.4)	78 (22.2)	47 (20.2)		
Marital status				0.046	0.830
Currently unmarried	477 (81.5)	288 (81.8)	189 (81.1)		
Currently married	108 (18.5)	64 (18.2)	44 (18.9)		
Whether having kids				0.878	0.349
No	443 (75.9)	271 (77.2)	172 (73.8)		
Yes	141 (24.1)	80 (22.8)	61 (26.2)		
Whether having medical insurance				0.460	0.497
Yes	512 (87.8)	310 (88.6)	202 (86.7)		
No	71 (12.2)	40 (11.4)	31 (13.3)		
ART medication knowledge score	Mean = 10.32, SD = 1.50	Mean = 10.26, SD = 1.52	Mean = 10.41, SD = 1.46	− 1.160	0.247
Sex role				0.805	0.669
Insertive role	131 (22.9)	75 (21.8)	56 (24.5)		
Receptive role	170 (29.7)	106 (30.8)	64 (27.9)		
Both	272 (47.5)	163 (47.4)	109 (47.6)		
Sex orientation				6.135	0.047
Homosexual	351 (60.0)	197 (56.0)	154 (66.1)		
Bisexual	156 (26.7)	102 (29.0)	54 (23.2)		
Heterosexual or unclear	78 (13.3)	53 (15.1)	25 (10.7)		
Sex orientation disclosure				1.481	0.224
No	259 (44.3)	163 (46.3)	96 (41.2)		
Yes	326 (55.7)	189 (53.7)	137 (58.8)		
HIV status disclosure				3.048	0.081
No	221 (37.8)	143 (40.6)	78 (33.5)		
Yes	364 (62.2)	209 (59.4)	155 (66.5)		
Alcohol use in the past 12 months				0.233	0.629
No	244 (41.7)	144 (40.9)	100 (42.9)		
Yes	341 (58.3)	208 (59.1)	133 (57.1)		
Ever drug use				2.779	0.095
No	547 (93.5)	334 (94.9)	213 (91.4)		
Yes	38 (6.5)	18 (5.1)	20 (8.6)		
Having condomless anal sex in the past 3 months				0.298	0.585
No	523 (90.5)	313 (89.9)	210 (91.3)		
Yes	55 (9.5)	35 (10.1)	20 (8.7)		

Data are presented as no. (%) unless otherwise indicated. Missing values were not taken into account in the percentage calculation

ART, antiretroviral therapy; SD, standard deviation; CNY, Chinese Yuan (1 CNY = 0.1534 USD)

As shown in Table 2, almost all of the participants (94.7%) had received adherence education before ART initiation. More than half of participants (58.0%) took ART medications once a day. The majority (91.6%) had medication reminders. Only 29.2% of participants reported ever missing doses before this survey but 61.9% reported side effects during ART. Compared with those in long-term ART group, participants in short-term group were significantly more likely to initiate ART in the same year when HIV diagnosed (87.5% vs. 78.1%, *P* = 0.003) and reported complete ART adherence (79.8% vs. 69.1%, *P* = 0.003).

**Table 2:** Comparison of ART-related information among MSM living with HIV on short-term and long-term ART in Jinan of China (N = 585)

Variables	Total (N = 585)	Short-term ART group (n = 352)	Long-term ART group (n = 233)	χ^2^/t	*P*-value
Intervals between HIV diagnosis and ART initiation				9.085	0.003
In the same year	490 (83.8)	308 (87.5)	182 (78.1)		
More than 1 year	95 (16.2)	44 (12.5)	51 (21.9)		
Frequency of taking medications				5.956	0.015
Once a day	339 (58.0)	218 (62.1)	121 (51.9)		
Twice a day	245 (42.0)	133 (37.9)	112 (48.1)		
Types of ART medication				6.817	0.009
Two or one	46 (7.9)	36 (10.2)	10 (4.3)		
Three	539 (92.1)	316 (89.8)	223 (95.7)		
Distance of medical visit				14.402	< 0.001
≤ 20 km	286 (49.5)	195 (55.9)	91 (39.7)		
> 20 km	292 (50.5)	154 (44.1)	138 (60.3)		
Adherence education before ART initiation				0.258	0.612
Yes	554 (94.7)	332 (94.3)	222 (95.3)		
No	31 (5.3)	20 (5.7)	11 (4.7)		
Having medication reminders				1.149	0.284
Yes	536 (91.6)	319 (90.6)	217 (93.1)		
No	49 (8.4)	33 (9.4)	16 (6.9)		
Ever missing doses				4.091	0.043
No	414 (70.8)	260 (73.9)	154 (66.1)		
Yes	171 (29.2)	92 (26.1)	79 (33.9)		
Report side effect while taking medications				0.100	0.752
No	223 (38.1)	136 (38.6)	87 (37.3)		
Yes	362 (61.9)	216 (61.4)	146 (62.7)		
HIV treatment self-efficacy score	Mean = 114.25, SD = 15.50	Mean = 114.71, SD = 14.01	Mean = 113.56, SD = 17.53	0.878	0.380
ART adherence in the past month				8.741	0.003
Suboptimal	143 (24.4)	71 (20.2)	72 (30.9)		
Complete	442 (75.6)	281 (79.8)	161 (69.1)		

Data are presented as no. (%) unless otherwise indicated. Missing values were not taken into account in the percentage calculation

ART, antiretroviral therapy; SD, standard deviation; CNY, Chinese Yuan (1 CNY = 0.1534 USD)

More information about previous missing doses, ART side effect, adherence education before ART initiation, and medication reminders was detailed in Additional file 1: Tables S1–S4.

### Impact of ART duration on ART adherence

In the univariate regression, in addition to ART duration, variables including education, ART medication knowledge score, sex role, sex orientation, sex orientation disclosure, alcohol use, condomless anal sex, intervals between HIV diagnosis and ART initiation, frequency of taking medications, and whether having medication reminders were included in further multivariable analysis (Table 3).

**Table 3:** Factors associated with ART adherence in univariate and multivariable regression among MSM living with HIV in Jinan of China (N = 585)

Variables	Univariate regression		Multivariate regression	
	Crude OR (95% CI)	*P*-value	AOR (95% CI)	*P*-value
Duration of ART (years)	0.89 (0.83–0.96)	0.002	0.88(0.81–0.95)	0.001
Age	0.99 (0.97–1.02)	0.564		
Living area				
Urban area	Ref.			
Rural area or county town	1.08 (0.56–2.20)	0.827		
Education				
High school and below	Ref.			
College and above	1.40 (0.96–2.06)	0.082		
Employment				
Yes	Ref.			
No	1.14 (0.57–2.50)	0.718		
Monthly income (CNY)				
≤ 5000	Ref.			
5001–8000	0.86 (0.56–1.33)	0.501		
> 8000	0.97 (0.60–1.61)	0.911		
Marital status				
Currently unmarried	Ref.			
Currently married	0.86 (0.54–1.39)	0.591		
Whether having kids				
No	Ref.			
Yes	0.80 (0.53–1.24)	0.315		
Whether having medical insurance				
Yes	Ref.			
No	0.68 (0.40–1.19)	0.167		
ART medication knowledge score	1.19 (1.06–1.35)	0.004	1.20(1.05–1.37)	0.009
Sex role				
Insertive role	Ref.			
Receptive role	1.76 (1.03–3.03)	0.039		
Both	1.16 (0.72–1.83)	0.539		
Sex orientation				
Homosexual	Ref.		Ref.	
Bisexual	0.69 (0.45–1.05)	0.079	0.57(0.36–0.92)	0.020
Heterosexual or unclear	1.52 (0.82–3.02)	0.200	1.30(0.64–2.85)	0.484
Sex orientation disclosure				
No	Ref.		Ref.	
Yes	0.60 (0.40–0.88)	0.010	0.60(0.39–0.92)	0.021
HIV status disclosure				
No	Ref.			
yes	0.82 (0.55–1.21)	0.319		
Alcohol use in the past 12 months				
No	Ref.		Ref.	
Yes	0.49 (0.33–0.74)	< 0.001	0.44(0.28–0.67)	< 0.001
Ever drug use				
No	Ref.			
Yes	0.60 (0.30–1.24)	0.151		
Having condomless anal sex in the past 3 months				
No	Ref.		Ref.	
Yes	0.58 (0.33–1.07)	0.073	0.58(0.31–1.11)	0.094
Intervals between HIV diagnosis and ART initiation				
In the same year	Ref.			
More than 1 year	0.58 (0.36–0.94)	0.023		
Frequency of taking medications				
Once a day	Ref.		Ref.	
Twice a day	0.66 (0.45–0.97)	0.032	0.71(0.47–1.08)	0.112
Types of ART medication				
Two or one	Ref.			
Three	0.81 (0.42–1.63)	0.531		
Distance of medical visit				
≤ 20 km	Ref.			
> 20 km	0.84 (0.57–1.22)	0.356		
Adherence education before ART initiation				
Yes	Ref.			
No	0.66 (0.31–1.50)	0.301		
Having medication reminders				
Yes	Ref.		Ref.	
No	0.33 (0.18–0.60)	< 0.001	0.27(0.14–0.52)	< 0.001
Having side effects while taking medications				
No	Ref.			
Yes	1.11 (0.76–1.65)	0.586		
HIV treatment self-efficacy score	1.01 (0.99–1.02)	0.166		

ART, antiretroviral therapy; CNY, Chinese Yuan (1 CNY = 0.1534 USD); OR, odds ratio; AOR, adjusted odds ratio; CI, confidence interval

In multivariable regression, those with longer ART duration (AOR = 0.88, 95% CI 0.81–0.95), having disclosed their sex orientation (AOR = 0.60, 95% CI 0.39–0.92), reporting alcohol use in the past 12 months (AOR = 0.44, 95% CI 0.28–0.67), and those who did not have medication reminders (AOR = 0.27, 95% CI 0.14–0.52) were less likely to report complete ART adherence. Compared with homosexual participants, bisexual counterparts (AOR = 0.57, 95% CI 0.36–0.92) were less likely to report complete ART adherence. Those with higher ART medication knowledge score (AOR = 1.20, 95% CI 1.05–1.37) were more likely to report complete ART adherence (Table 3).

### Trend analysis of complete ART adherence

**Fig. 1:**
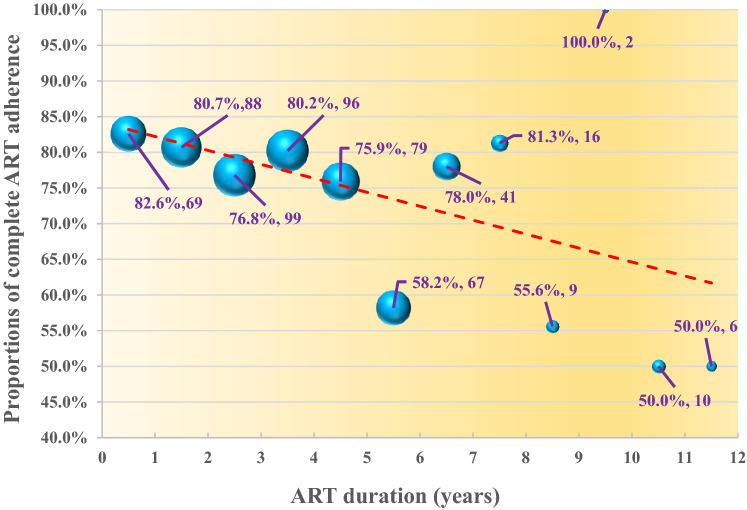
Trend of proportion of PLWH with complete adherence based on different ART durations (N = 582). *Note*: There was one participant initiating ART in 2003, 2005 and 2006 (i.e. 14, 15 and 17 years of ART duration) respectively. We excluded the three participants to Mann–Kendall test due to insufficient sample size. The size of bubble indicated the number of participants (i.e. denominator) based on different ART duration. ART, antiretroviral therapy

The proportions of PLWH with complete ART adherence was 75.6%, 79.8% and 69.1% for all participants, in short-term ART group and in long-term ART group respectively. The proportions of PLWH with complete ART adherence and the number of participants (i.e. denominator) based on different ART durations (0–11 years from 2009 to 2020) were displayed in Fig. 1. One-side Mann–Kendall test showed that there was a significantly descending trend (Z = 1.787, *P* = 0.037).

### Subgroup analysis

**Fig. 2:**
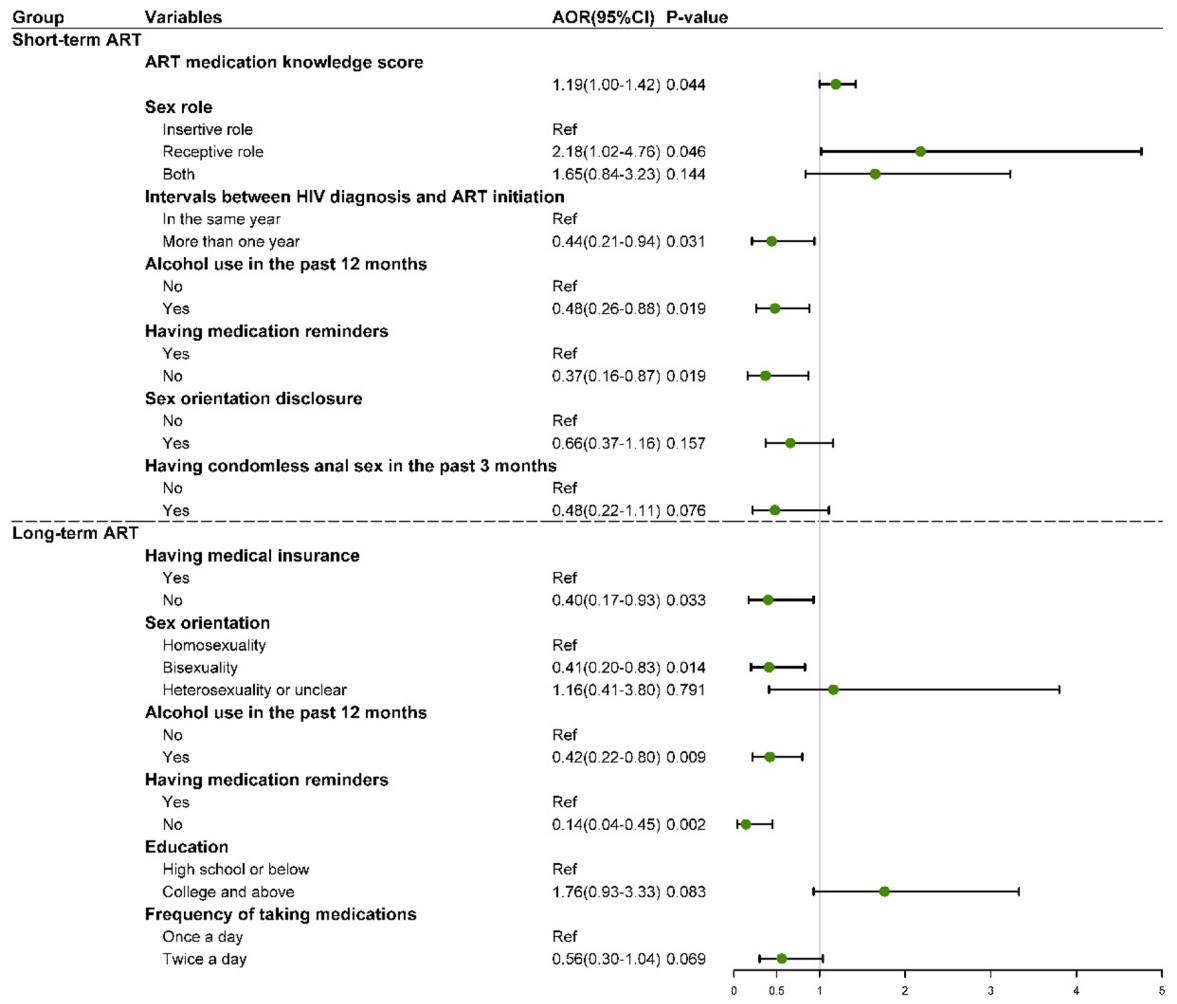
Factors associated with ART adherence in multivariable regression among MSM living with HIV on short-term ART and long-term ART in Jinan of China (N = 585). ART, antiretroviral therapy; AOR, adjusted odds ratio; CI, confidence interval

The subgroup analysis based on ART duration was displayed in Additional file 1: Table S5 (univariate regressions) and Fig. 2 (multivariable regressions). Results showed that alcohol use in the past 12 months and lack of medication reminders were barriers to complete adherence for both short-term and long-term ART group. Besides, for short-term ART group, those with delayed ART initiation (more than 1 year) were less likely to report complete ART adherence, while those who were receptive role (compared with insertive role) and having higher ART medication knowledge score were more likely to report complete ART adherence. For long-term ART group, those without medical insurance and bisexual men were less likely to report complete ART adherence.

## Discussion

In this study, we investigated the impact of ART duration on ART adherence among MSM living with HIV in Jinan of China. Simultaneously, we identified the trend of proportion of PLWH with complete adherence based on different ART durations. In addition, we explored and compared factors associated with ART adherence between short-term and long-term ART group.

This study showed that participants with longer ART duration were less likely to report complete adherence, which was consistent with studies in Europe and US [10–13] but inconsistent with a cohort study in Asia and Africa [8]. However, this cohort study mainly included Southeast Asian countries where ART polices were different from China and measured adherence using visual analogue scale without considering adherence to dosing schedule. Our findings may be because the longer the ART duration, the more likely the patients feel “tired” for the treatment. Further, long-term asymptomatic status may make some patients doubt whether it is necessary to maintain complete adherence. Studies have shown that higher current CD4 level was correlated with higher likelihood of non-adherence to ART among patients on long-term ART, which indicated the patients’ patience to maintain complete adherence decreased over time [19, 20]. In addition, NFATP in China scaled up rapidly and China entered the era of “Treat all” in 2016. Evidence showed that early initiation of ART regardless of CD4 level can lead to higher likelihood of adherence [21]. Further, our study found that more participants on short-term ART received pre-ART adherence education via Internet (e.g., WeChat or QQ) (see Additional file 1: Table S3). These digital platforms can significantly improve interactions between patients and healthcare providers, which can effectively enhance medication self-efficacy and decrease medication-taking difficulties [22]. Our findings indicated the importance of sustained efforts to emphasize continued adherence over time, especially for those on long-term ART.

The proportions of complete ART adherence in the past month was 75.6% in this study, which was lower than a meta-analysis in China (80.9%, 95% CI 74.7–85.9%) [5]. This may be due to different methods of measuring adherence. Most studies only focused on missing doses and few evaluated non-adherence to dosing schedule. However, complete ART adherence involves not only avoiding missing doses, but also taking the pills at the right time and in the right way. Taking ART medications at irregular times can change its pharmacokinetic and pharmacodynamics effects, which may exacerbate the risk of treatment failure and drug resistance [23]. Obviously, non-adherence to dosing schedule should not be neglected.

We found that alcohol use was a key barrier to complete ART adherence, which was consistent with many studies demonstrating the harmful effects of alcohol use on ART adherence and other HIV-related outcomes [24–26]. Lack of medication reminder was another important barrier for both of the groups. It was noteworthy that forgetting was the most frequently cited reason of ever missing doses (see Additional file 1: Table S1). Challenges related to forgetfulness can be overcome through providing reminder services. Text messaging have been widely used and recommended by WHO for its convenience and cost-effectiveness [27, 28].

Studies have evaluated the association between HIV status disclosure and ART adherence, but few explored the effect of sex orientation disclosure on ART adherence among MSM living with HIV [29–31]. This study found that individuals who disclosed their sex orientation were less likely to report complete adherence. Traditionally, MSM were not acceptable in Chinese society even before the HIV infection [32, 33]. Disclosure of MSM orientation may result in the increase of psychological pressure, which was reported a key barrier to ART adherence [6]. In China, sex orientation disclosure might be even more difficult than HIV status disclosure among MSM living with HIV. Bisexual men were less likely to report complete ART adherence compared with homosexual men. This phenomenon was also observed in long-term ART subgroup. In China, adults are expected to get married at a certain age. Different from homosexual men, bisexual men with long-term ART were more likely to eventually get married with heterosexual women, which may increase anxiety and stress to keep the secrets from family members [17, 34]. In short-term ART group, MSM who were receptive role were more likely to report complete adherence compared with insertive role. It was reported that HIV-positive gay men usually had one or more sexual problems (e.g. erectile dysfunction) [18] and tended to be receptive role. Consequently, they may be more likely to report complete adherence to acheive viral suppression and reduce their partners’ risk of HIV acquisition. Physiological and psychological differences between receptive and insertive MSM may also influence the interpretations of this finding. In-depth interviews are needed to better reveal the phenomenon.

This study has important public health implications. First, the study indicated the importance of sustained efforts to emphasize continued adherence over time, especially for those on long-term ART. Digital platforms (e.g., WeChat and QQ) can be utilized to improve healthcare interactions. Second, the focus should be on individuals who use alcohol and do not have medication reminders. Alcohol screening and text messaging reminder services can be integrated into routine management of PLWH. Third, subgroup analysis showed that factors associated with ART adherence were not completely consistent between short-term and long-term ART group, which indicated the necessity of tailored intervention. In addition, future qualitative studies need to be conducted to further understand the psychosocial mechanism of the assocations between sexual characteristics (e.g., sex role and sex orientation) and adherence among MSM.

Although some important findings are revealed in this study, there are also limitations. First, ART adherence was measured through self-report instead of more objective measurement such as electronic monitoring, CD4 T-cell counts and HIV viral load. Due to cost and timeline, we did not collect biological samples. However, evidence showed self-report adherence was strongly associated with HIV viral load [35]. Second, the findings may not be generalizable to all MSM living with HIV because the participants were recruited from one hospital in Jinan using convenience sampling. Finally, adherence is a complex dynamic phenomenon influenced by individual and contextual factors. Unobserved factors may confound the impact of ART duration on adherence. A long-term cohort in Chinese context is needed to monitor ART adherence in the future.

## Conclusions

In conclusion, MSM living with HIV with longer ART duration were less likely to report complete adherence. A descending trend of proportions of PLWH with complete adherence based on different ART durations was identified. Factors associated with ART adherence between short-term and long-term ART group were not completely consistent. Sustained efforts to encourage maintaining complete adherence for a lifetime are necessary, especially for those on long-term ART. Future adherence interventions should be tailored to subgroups with different ART durations and individuals with specific characteristics, especially for those who report alcohol use, do not have medication reminders and bisexual men on long-term ART.

## Supplementary Information

**Additional file 1. Table S1**: Comparison of information about ART missing doses among MSM living with HIV on short-term and long-term ART in Jinan of China (N = 171). **Table S2**: Comparison of information about ART side effects among MSM living with HIV on short-term and long-term ART in Jinan of China (N = 361). **Table S3**: Comparison of information about pre-ART adherence education among MSM living with HIV on short-term and long-term ART in Jinan of China (N = 550). **Table S4**: Comparison of channels of medication reminders among MSM living with HIV on short-term and long-term ART in Jinan of China (N = 585). **Table S5**: Factors associated with ART adherence in univariate regression among MSM living with HIV on short-term ART and long-term ART in Jinan of China (N = 585).

## Data Availability

The datasets used and/or analysed during the current study are available from the corresponding author (Wei Ma, weima@sdu.edu.cn) on reasonable request.

## Abbreviations

AOR: Adjusted odds ratio
ART: Antiretroviral therapy
CI: Confidence interval
CNY: Chinese Yuan
MSM: Men who have sex with men
NFATP: National Free Antiretroviral Treatment Program
PLWH: People living with HIV
STI: Sexually transmitted infections
USD: United States dollar
