# Supplementary material for: Impact of antiretroviral therapy (ART) duration on ART adherence among men who have sex with men (MSM) living with HIV in Jinan of China
Source: AIDS Res Ther. 2022 Nov 24;19:55. doi: 10.1186/s12981-022-00482-z (PMC9694540; doi:10.1186/s12981-022-00482-z)
Supplement: Supplementary file 1 — Additional file 1. Table S1: Comparison of information about ART missing doses among MSM living with HIV on short-term and long-term ART in Jinan of China (N = 171). Table S2: Comparison of information about ART side effects among MSM living with HIV on short-term and long-term ART in Jinan of China (N = 361). Table S3: Comparison of information about pre-ART adherence education among MSM living with HIV on short-term and long-term ART in Jinan of China (N = 550). Table S4: Comparison of channels of medication reminders among MSM living with HIV on short-term and long-term ART in Jinan of China (N = 585). Table S5: Factors associated with ART adherence in univariate regression among MSM living with HIV on short-term ART and long-term ART in Jinan of China (N = 585). [file 12981_2022_482_MOESM1_ESM.docx]

**Additional file**

**Table S1. Comparison of information about ART missing doses among MSM living with HIV on short-term and long-term ART in Jinan of China (N=171)**

| **Variables** | **Total**  **(N=171)** | **Short-term ART group (n=92)** | **Long-term ART group (n=79)** | **P-value^†^** |  |
| --- | --- | --- | --- | --- | --- |
| **Time of last missing doses** |  |  |  | 0.482 |  |
| Within the past week | 22(12.9) | 12(13.0) | 10(12.7) |  |  |
| 1-2 weeks ago | 16(9.4) | 7(7.6) | 9(11.4) |  |  |
| 3-4 weeks ago | 16(9.4) | 6(6.5) | 10(12.7) |  |  |
| 1-3 months ago | 31(18.1) | 16(17.4) | 15(19.0) |  |  |
| 3 months ago | 86(50.3) | 51(55.4) | 35(44.3) |  |  |
| **Frequency of missing doses** |  |  |  | 0.426 |  |
| Less than once a week on average | 5(2.9) | 2(2.2) | 3(3.8) |  |  |
| 1-2 weeks on average | 4(2.3) | 1(1.1) | 3(3.8) |  |  |
| 3-4 weeks on average | 6(3.5) | 2(2.2) | 4(5.1) |  |  |
| 1-3 months on average | 28(16.4) | 18(19.6) | 10(12.7) |  |  |
| More than 3 months on average | 128(74.9) | 69(75.0) | 59(74.7) |  |  |
| **Schedules of missing doses in usual** |  |  |  | 0.271 |  |
| Morning ( awake to 12:00 am) | 67(39.2) | 31(33.7) | 36(45.6) |  |  |
| Afternoon (12:00 am to 5:00 pm) | 5(2.9) | 2(2.2) | 3(3.8) |  |  |
| Evening (5:00 pm to 9:00 pm) | 10(5.8) | 5(5.4) | 5(6.3) |  |  |
| Night (9:00 pm to awake) | 89(52.0) | 54(58.7) | 35(44.3) |  |  |
| **Reasons of missing doses** |  |  |  | 0.146 |  |
| Side effects | 3(1.8) | 1(1.1) | 2(2.6) |  |  |
| Pill burden (Too many pills to take) | 0(0.0) | 0(0.0) | 0(0.0) |  |  |
| Forgetfulness | 111(64.9) | 58(63.0) | 53(67.1) |  |  |
| No medications left | 2(1.2) | 2(2.2) | 0(0.0) |  |  |
| No need to take medications | 0(0.0) | 0(0.0) | 0(0.0) |  |  |
| Business travel | 41(24.1) | 26(28.3) | 15(19.2) |  |  |
| Fear of privacy disclosure | 16(9.4) | 11(12.0) | 5(6.4) |  |  |
| Having difficulty in taking medications in specific time (e.g. on an empty stomach or at meal) | 17(10.0) | 5(5.4) | 12(15.4) |  |  |
| Not sure how to take medications | 0(0.0) | 0(0.0) | 0(0.0) |  |  |
| Other reasons | 11(6.5) | 6(6.5) | 5(6.4) |  |  |
| Note: Data are presented as no. (%).  Abbreviations: ART, antiretroviral therapy  ^†^ The P-values were calculated using Chi-square tests or Fisher’s exact tests | | | | | |

**Table S2. Comparison of information about ART side effects among MSM living with HIV on short-term and long-term ART in Jinan of China (N=361)**

| **Variables** | **Total**  **(N=361)** | **Short-term ART group (n=216)** | **Long-term ART group (n=145)** | **P-value^†^** |  |
| --- | --- | --- | --- | --- | --- |
| **Side effects reported while taking medications** |  |  |  | 0.568 |  |
| Dizziness and headache | 178(49.3) | 104(48.1) | 74(51.0) |  |  |
| Gastric reactions (e.g., nausea, vomiting) and intestinal reactions (e.g., abdominal pain, diarrhea) | 98(27.1) | 60(27.8) | 38(26.2) |  |  |
| Liver and kidney damage | 54(15.0) | 26(12.0) | 28(19.3) |  |  |
| Fever | 34(9.4) | 23(10.6) | 11(7.6) |  |  |
| Weakness/sleepiness | 137(38.0) | 83(38.4) | 54(37.2) |  |  |
| Insomnia/dreaminess | 143(39.6) | 86(39.8) | 57(39.3) |  |  |
| Rash | 84(23.3) | 53(24.5) | 31(21.4) |  |  |
| Mental disorders (e.g., depression) | 48(13.3) | 26(12.0) | 22(15.2) |  |  |
| Other side effects | 16(4.4) | 7(3.2) | 9(6.2) |  |  |
| **Measures to deal with side effects** |  |  |  | 0.812 |  |
| Take no measures | 123(34.1) | 71(32.9) | 52(35.9) |  |  |
| Stop taking medications | 0(0.0) | 0(0.0) | 0(0.0) |  |  |
| Seek a medical visit | 162(44.9) | 97(44.9) | 65(44.8) |  |  |
| Seek help from physicians through calling or sending messages | 78(21.6) | 47(21.8) | 31(21.4) |  |  |
| Surf the Internet for related information | 51(14.1) | 34(15.7) | 17(11.7) |  |  |
| Other measures | 8(2.2) | 4(1.9) | 4(2.8) |  |  |
| Note: Data are presented as no. (%)  Abbreviations: ART, antiretroviral therapy  ^†^ The P-values were calculated using Chi-square tests or Fisher’s exact tests | | | | | |

**Table S3. Comparison of information about pre-ART adherence education among MSM living with HIV on short-term and long-term ART in Jinan of China (N=550)**

| **Variables** | **Total (N=550)** | **Short-term ART group (n=329)** | **Long-term ART group (n=221)** | **P-value^†^** |  |
| --- | --- | --- | --- | --- | --- |
| **Practitioners of adherence education** |  |  |  | 0.543 |  |
| CBOs | 108(19.6) | 70(21.3) | 38(17.2) |  |  |
| Community health service centers | 26(4.7) | 17(5.2) | 9(4.1) |  |  |
| Medical staff in hospital | 391(71.1) | 243(73.9) | 148(67.0) |  |  |
| CDC | 372(67.6) | 217(66.0) | 155(70.1) |  |  |
| Others | 5(0.9) | 2(0.6) | 3(1.4) |  |  |
| **Channels of adherence education** |  |  |  | 0.526 |  |
| Face-to-face communication | 469(85.3) | 275(83.6) | 194(87.8) |  |  |
| Telephone | 148(26.9) | 88(26.7) | 60(27.1) |  |  |
| Brochure | 85(15.4) | 54(16.4) | 31(14.0) |  |  |
| Internet (e.g. WeChat and QQ) | 136(24.7) | 90(27.4) | 46(20.8) |  |  |
| Other channels | 4(0.7) | 2(0.6) | 2(0.9) |  |  |
| Note: Data are presented as no. (%)  Abbreviations: ART, antiretroviral therapy; CBO, community-based organization; CDC, Center for Disease Control and Prevention  ^†^ The P-values were calculated using Chi-square tests or Fisher’s exact tests | | | | | |

**Table S4. Comparison of channels of medication reminders among MSM living with HIV on short-term and long-term ART in Jinan of China (N=585)**

| **Variables** | **Total**  **(N=585)** | **Short-term ART group (n=352)** | **Long-term ART group (n=233)** | **P-value^†^** |  |
| --- | --- | --- | --- | --- | --- |
| **Channels of medication reminders** |  |  |  | 0.391 |  |
| No reminders | 49(8.4) | 33(9.4) | 16(6.9) |  |  |
| Cellphone alarms | 521(89.1) | 312(88.6) | 209(89.7) |  |  |
| Calendar | 4(0.7) | 3(0.9) | 1(0.4) |  |  |
| Family members or friends | 3(0.5) | 1(0.3) | 2(0.9) |  |  |
| Other channels | 8(1.4) | 3(0.9) | 5(2.1) |  |  |
| Note: Data are presented as no. (%)  Abbreviations: ART, antiretroviral therapy  ^†^ The P-value was calculated using Chi-square tests or Fisher’s exact tests | | | | | |

**Table S5. Factors associated with ART adherence in univariate regression among MSM living with HIV on short-term ART and long-term ART in Jinan of China (N=585)**

|  | **Short-term ART group** | | **Long-term ART group** | |
| --- | --- | --- | --- | --- |
| **Variables** | **OR(95%CI)** | **P-value** | **OR(95%CI)** | **P-value** |
| **Age** | 1.01(0.98-1.04) | 0.710 | 1.00(0.96-1.03) | 0.776 |
| **Living area** |  |  |  |  |
| Urban area | Ref |  | Ref |  |
| Rural area or county town | 1.40(0.56-4.26) | 0.503 | 0.80(0.31-2.22) | 0.656 |
| **Education** |  |  |  |  |
| High school and below | Ref |  | Ref |  |
| College and above | 1.00(0.58-1.70) | 0.994 | 2.04(1.16-3.61) | 0.014 |
| **Employment** |  |  |  |  |
| Yes | Ref |  | Ref |  |
| No | 1.01(0.44-2.61) | 0.979 | 1.05(0.28-4.96) | 0.950 |
| **Monthly income (CNY)** |  |  |  |  |
| ≤5000 | Ref |  | Ref |  |
| 5001-8000 | 0.58(0.32-1.06) | 0.072 | 1.49(0.79-2.84) | 0.218 |
| >8000 | 0.79(0.40-1.57) | 0.483 | 1.27(0.61-2.72) | 0.534 |
| **Marital status** |  |  |  |  |
| Currently unmarried | Ref |  | Ref |  |
| Currently married | 0.99(0.52-2.01) | 0.975 | 0.74(0.37-1.49) | 0.385 |
| **Whether having kids** |  |  |  |  |
| No | Ref |  | Ref |  |
| Yes | 0.84(0.46-1.56) | 0.565 | 0.80(0.43-1.51) | 0.488 |
| **Whether having medical insurance** |  |  |  |  |
| Yes | Ref |  | Ref |  |
| No | 1.20(0.54-3.07) | 0.675 | 0.42(0.19-0.91) | 0.027 |
| **ART medication knowledge score** | 1.17(1.00-1.38) | 0.044 | 1.26(1.04-1.54) | 0.018 |
| **Sex role** |  |  |  |  |
| Insertive role | Ref |  | Ref |  |
| Receptive role | 2.19(1.06-4.61) | 0.036 | 1.31(0.58-2.94) | 0.513 |
| Both | 1.53(0.81-2.87) | 0.186 | 0.81(0.40-1.61) | 0.561 |
| **Sex orientation** |  |  |  |  |
| Homosexual | Ref |  | Ref |  |
| Bisexual | 0.88(0.49-1.58) | 0.652 | 0.44(0.23-0.83) | 0.011 |
| Heterosexual or unclear | 1.43(0.65-3.50) | 0.394 | 1.50(0.57-4.73) | 0.446 |
| **Sex orientation disclosure** |  |  |  |  |
| No | Ref |  | Ref |  |
| Yes | 0.61(0.35-1.03) | 0.069 | 0.62(0.34-1.10) | 0.104 |
| **HIV status disclosure** |  |  |  |  |
| No | Ref |  | Ref |  |
| yes | 1.01(0.59-1.71) | 0.966 | 0.68(0.37-1.24) | 0.219 |
| **Alcohol use in the past 12 months** |  |  |  |  |
| No | Ref |  | Ref |  |
| Yes | 0.50(0.28-0.86) | 0.016 | 0.47(0.26-0.84) | 0.012 |
| **Ever drug use** |  |  |  |  |
| No | Ref |  | Ref |  |
| Yes | 0.64(0.23-2.05) | 0.412 | 0.64(0.25-1.71) | 0.360 |
| **Having condomless anal sex in the past 3 months** |  |  |  |  |
| No | Re |  | Ref |  |
| Yes | 0.52(0.25-1.15) | 0.092 | 0.64(0.25-1.71) | 0.358 |
| **Intervals between HIV diagnosis and ART initiation** |  |  |  |  |
| In the same year | Ref |  | Ref |  |
| More than one year | 0.43(0.22-0.87) | 0.016 | 0.87(0.45-1.71) | 0.671 |
| **Frequency of taking medications** |  |  |  |  |
| Once a day | Ref |  | Ref |  |
| Twice a day | 0.92(0.54-1.58) | 0.764 | 0.51(0.29-0.89) | 0.018 |
| **Types of pills** |  |  |  |  |
| Two or one | Ref |  | Ref |  |
| Three | 0.62(0.29-1.42) | 0.233 | 1.05(0.28-4.96) | 0.950 |
| **Distance of medical visit** |  |  |  |  |
| ≤20 kilometers | Ref |  | Ref |  |
| >20 kilometers | 0.99(0.59-1.69) | 0.976 | 0.83(0.46-1.47) | 0.518 |
| **Adherence education before ART initiation** |  |  |  |  |
| Yes | Ref |  | Ref |  |
| No | 0.74(0.28-2.35) | 0.581 | 0.52(0.15-1.85) | 0.292 |
| **Having medication reminders** |  |  |  |  |
| Yes | Ref |  | Ref |  |
| No | 0.40(0.19-0.87) | 0.018 | 0.18(0.05-0.51) | 0.002 |
| **Report side effects while taking medications** |  |  |  |  |
| No | Ref |  | Ref |  |
| Yes | 0.96(0.56-1.65) | 0.877 | 1.32(0.74-2.39) | 0.351 |
| **HIV treatment self-efficacy score** | 1.01(0.99-1.03) | 0.283 | 1.01(0.99-1.02) | 0.450 |
| Note: Abbreviations: ART, antiretroviral therapy; CNY, Chinese Yuan (1 CNY=0.1534 USD); OR, odds ratio; CI, confidence interval | | | | |
